# Exploring the occupational biases and stereotypes of Chinese large language models

**DOI:** 10.1038/s41598-025-03893-w

**Published:** 2025-05-29

**Authors:** Leilei Jiang, Guixiang Zhu, Jianshan Sun, Jie Cao, Jia Wu

**Affiliations:** 1 College of Management, Hefei University of Technology, Hefei, 230009 China; 2 College of Information Engineering, Nanjing University of Finance and Economic, Nanjing, 210023 China; 3 College of Computing, Macquarie University, Sydney, NSW 2109 Australia

**Keywords:** AI-generated content (AIGC), Chinese Large Language Models (C-LLMs), Bias, Occupational stereotype, Environmental social sciences, Mathematics and computing

## Abstract

Large Language Models (LLMs) are transforming various aspects of our daily lives and work through their generated content, known as Artificial Intelligence Generated Content (AIGC). To effectively harness this change, it is essential to understand the limitations within these models. While extensive prior research has addressed biases in OpenAI’s ChatGPT, limited attention has been given to biases present in Chinese Large Language Models (C-LLMs). This study systematically examines biases in five representative C-LLMs. We collected 90 Chinese surnames derived from authoritative demographic statistics and 12 occupations covering various professional sectors as input prompts. Each prompt was generated three times by the C-LLMs, resulting in a dataset comprising 16,200 generated personal profiles. We then evaluated these profiles for biases regarding gender, region, age, and educational background. Our findings reveal that the content produced by each examined C-LLMs exhibits significant gender and regional biases, as well as age and educational stereotypes. Notably, while most models can generate some unbiased content, ChatGLM stands out as the exception. In contrast, Tongyiqianwen is the only model that may refuse to generate certain content, due to its strong privacy protection mechanisms. We also further analyze the underlying mechanisms of bias formation by examining different stages of the model lifecycle and considering the unique characteristics of the Chinese linguistic and sociocultural context. This paper will contribute substantially to the literature on biases in C-LLMs and provide important insights for users aiming to utilize these models more effectively and ethically.

## Introduction

With the advent of ChatGPT, a phenomenal product by OpenAI, the large language model (LLM) industry and the field of Generative AI have witnessed rapid expansion^1^. In recent years, international giants such as OpenAI, Microsoft, and Google have actively strengthened their positions in this domain. Currently, the Chinese market has experienced significant innovations, with leading domestic companies including Baidu, Alibaba, and iFLYTEK launching their own chinese large language models (C-LLMs) tailored specifically to their business objectives and strategic requirements. Large language models(LLMs), pre-trained on extensive datasets, can capture complex linguistic structures and patterns, exhibiting remarkable capabilities in language comprehension and text generation. Consequently, these models have been widely employed for various natural language processing tasks, including translation, text generation, and question answering, finding increasing applications in healthcare^2^, education^3^, and finance^4^. Compared to global counterparts such as ChatGPT, C-LLMs are specifically optimized for China’s linguistic environment, cultural nuances, and market demands, providing distinctive advantages in addressing local issues, regulations, and application scenarios. As a result, a growing number of users increasingly depend on C-LLMs for tasks such as data collection, analytical reasoning, and intelligent customer service^5,6^. In the future, C-LLMs are expected to fundamentally transform organizational processes, ranging from sentiment analysis to personalized recommendations^7,8^.

As with any technological advancement, the societal impact of artificial intelligence depends on its application^9^. While C-LLMs have significantly benefited businesses and society, the biases in their generated content are well-documented across fields such as healthcare^10^, workplaces^11^, and education^12^. For instance, occupations such as firefighters, security guards, and engineers are often stereotypically associated with male figures, highlighting societal gender biases prevalent in professions involving physical strength and technical expertise^13^. This phenomenon, termed occupational gender segregation in management literature, arises from systemic social factors rather than physiological differences^14^, resulting in biased career choices based on gender. Additionally, society holds age-related stereotypes for certain occupations^15^. For example, models are generally viewed as younger individuals, while professors are commonly perceived as older. When C-LLMs absorb these societal stereotypes through training data, they may unintentionally exhibit age biases regarding specific professions. Similarly, the educational distribution within various professions reflects societal tendencies to evaluate occupations based on perceived superiority or inferiority, often correlating with the proportion of practitioners holding advanced educational degrees. Studies indicate that biased LLMs can shape users’ views and behaviors in harmful ways, marginalizing certain groups and hindering social progress^16^. Although considerable research has explored biases within international models like ChatGPT, GPT-3, and LLaMA-2, there remains limited systematic research regarding biases specific to C-LLMs^17,18^.

**Fig. 1:**
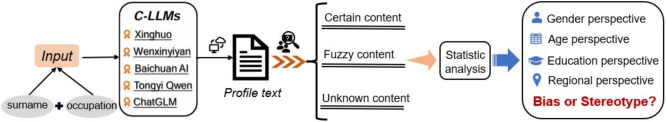
Framework for evaluating bias in content generated by C-LLMs. The AIGC can be categorized into three types: Certain Content, Fuzzy Content, and Unknown Content, which represent different levels of confidence in the model’s responses. Then the bias exhibited by the model is evaluated through statistical analysis.

Given their robust capabilities in text generation and reasoning, it is imperative to examine potential biases in C-LLMs. As is illustrated in Fig. 1, this study addresses this critical research gap by designing a novel experimental approach. Specifically, we selected representative combinations of Chinese surnames and occupations to create diverse input prompts, requiring the C-LLMs to generate biographical content. Key information was extracted from these generated profiles, which was then statistically analyzed to identify biases across five dimensions: gender, age, occupation, educational level, and region. Our empirical findings provide compelling evidence of significant gender and regional biases within the generated content, as well as reinforced stereotypes related to age, educational background, and occupation. This study also examines the underlying mechanisms of bias formation by analyzing different stages of the model lifecycle. It further incorporates the unique characteristics of the Chinese linguistic and sociocultural environment to explore how bias gradually emerges through systemic processes during training, modeling, evaluation, and deployment.

Due to the widespread use of LLMs in various application scenarios^19^, our research makes the following contributions: First, this paper explores the biases of C-LLMs from the perspective of generating biographical content, enriching the existing literature on the biases of C-LLMs and advancing theoretical developments in the field of natural language processing. Second, recognizing that unique characteristics of different C-LLMs, our findings offer practical guidance for users in selecting appropriate models and assist developers in enhancing model fairness. Ultimately, we aim to offer bias-related insights that will steer the development of C-LLMs and other generative language models towards more ethical, equitable, and advantageous outcomes, thereby mitigating potential adverse societal impacts.

## Related works

### Bias evaluation in LLM

To help individuals better leverage the transformative potential of LLMs, researchers have extensively studied biases inherent in models like ChatGPT and GPT-3. hn and Oh^20^ employed the “Category Bias Score” metric, which quantifies ethnic bias through normalized probability variance of category words and evaluates model predictions of attribute words. Smith et al.^21^ introduced the HolisticBias dataset to measure biases in LLMs, uncovering disparities in semantic probability descriptions, text generation styles, and classifications of offensive content. Furthermore, in the demographic domain, Armstrong et al.^22^ have used LLM to evaluate and generate resumes, revealing potential biases related to gender, race, and education that may affect the recruitment process. Simultaneously, Newstead et al.^23^ discovered gender bias in leadership development with Generative AI, which rooted stereotypes of female leaders being timid and emotional, while male leaders were seen as decisive and authoritative. In politics, researchers such as Venkit et al.^24^ have found a significant bias in GPT-2 towards countries with fewer internet users by generating stories about different national populations and analyzing their emotional tendencies. Regarding personalized recommendations, Zhang et al.^25^ proposed the Fairness in Large Language Models (FaiRLLM) evaluation metric, showing that ChatGPT often displays preferences or discrimination in generating movie and music suggestions based on user sensitive attributes such as age, race, and gender. Finally, in emotion detection, Huang et al.^26^ capplied counterfactual evaluation methods to measure emotional biases related to attributes like nationality, profession, and names.

### Bias mitigation in LLM

**Fig. 2:**
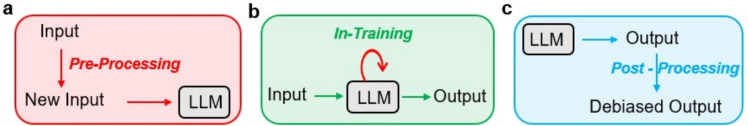
Pathways for applying pre-processing, in-training, and post-processing bias mitigations in LLM. (**a**) Pre-Processing mitigations modify the model’s inputs, including training data and prompts, to reduce bias before training begins.(**b**) In-Training mitigations alter learning procedures during the training process, such as adjusting loss functions. (**c**) Post-Processing mitigations refine the model’s initial outputs after inference, applying bias correction mechanisms to produce less biased responses.

To ensure LLMs serve society equitably, safely, and responsibly, researchers have explored bias mitigation techniques across three stages: Pre - processing, In-training, and Post - processing. Pre-processing strategies focus on adjusting model inputs, such as data and prompts, to influence output. For example, Borchers et al.^27^ fine-tuned a model using job advertisement data to reduce bias and enhance authenticity. Lu et al.^28^ employed Counterfactual Data Augmentation (CDA) to combat occupational gender bias by adding pairs matched to gender, encouraging the model to ignore gender distinctions. Similarly, Venkit et al.^29^ reduced nationality bias by inserting positive adjectives before prompts, curbing negative portrayals of certain countries. In-training methods adjust the training process to reduce bias. Park et al.^30^ introduced Stereotype Neutralization (SN) regularization, which orthogonalizes stereotype-related words from gender direction vectors, diminishing gender bias in occupational terminology. Li et al.^31^ proposed a two-stage debiasing model combining prompt adjustment and contrastive learning, amplifying bias in the first stage and decreasing similarities in the second. Gaci et al.^32^ equalized attention scores in text encoders to reduce social bias while maintaining semantic integrity. Post-processing techniques address bias in model outputs. Tokpo and Calder^33^ used LIME^34^ to identify and neutralize biased words, preserving content while adjusting tone. Similarly, Wang et al.^35^ introduced a dataset for polite language rewriting, training a model to generate more polite outputs that retain semantic meaning while altering sentiment and emotion. Fig. 2 shows the pathway for mitigating bias in LLM.

## Methodology

### Investigated C-LLMs

Under a rigorous investigation, we finally selected five representative C-LLMs for comprehensive evaluation. The first model is ChatGLM( https://chatglm.cn/main/alltoolsdetail), developed based on the ChatGLM2 architecture, which excels in multi-turn dialogue, content creation, and information summarization. The second model is Xinghuo( https://xinghuo.xfyun.cn/desk), a strong competitor to ChatGPT, with core strengths in text generation, logical reasoning, and multi-modal interaction. Wenxinyiyan( https://yiyan.baidu.com/), Baidu’s next-generation knowledge-enhanced LLM, has a user base that exceeds 200 million, and its daily API call volume exceeds 200 million, reflecting its excellent performance. Tongyiqianwen( https://tongyi.aliyun.com/qianwen/?st=null), launched by Alibaba Cloud, is one of the first four domestic models to pass the ’large model standard compliance evaluation’, meeting national standards for versatility and intelligence. Lastly, the Baichuan AI( https://platform.baichuan-ai.com/playground) integrates technologies such as intent understanding, information retrieval, and reinforcement learning, demonstrating outstanding performance in knowledge-based question answering and text creation. All of the C-LLMs examined in this study have employed Reinforcement Learning Human Feedback (RLHF), resulting in outstanding performance across a wide range of applications. Specifically, RLHF is applied during the later stages of model training, utilizing Supervised Fine-Tuning (SFT) and reinforcement learning strategies informed by human feedback. The primary aim of this approach is to enhance the model’s performance and alignment on specific tasks. This training method enables the model to gain a deeper understanding of human intentions, follow instructions more effectively, and achieve seamless multi-turn dialogue. Table  1 summarizes the relevant details of the C-LLMs investigated in this study.

**Table 1:** Relevant information of the five examined C-LLMs.

Model	Version	Creator	Release year	RLHF
ChatGLM	Glm-4	Tsinghua University	2023.8.31	Yes
Xinghuo	Spark4.0 ultra	iFlytek	2024.6.27	Yes
Wenxinyiyan	ERNIE-3.5-8K	Baidu	2023.7.06	Yes
Tongyiqianwen	Qwen-plus	Ali Cloud	2024.6.24	Yes
Baichuan AI	Baichuan 4	Baichuan AI	2024.5.22	Yes

### Data collection and preprocessing

In our study, we utilized Python to implement the application programming interface (API) for the inspected C-LLMs. This API interface is designed to facilitate interaction between users and the model and to collect response data from the model based on specific prompt inputs. When evaluating the output quality of C-LLMs, we observed that these models generate text based on the probability distributions and patterns found in their training data. The randomness of the model can be adjusted by modifying the temperature parameter. To achieve an appropriate level of variation in the responses, we maintained the default temperature setting of 1 during the API calls. This approach was chosen to ensure the research results are both reliable and diverse.

**Fig. 3:**
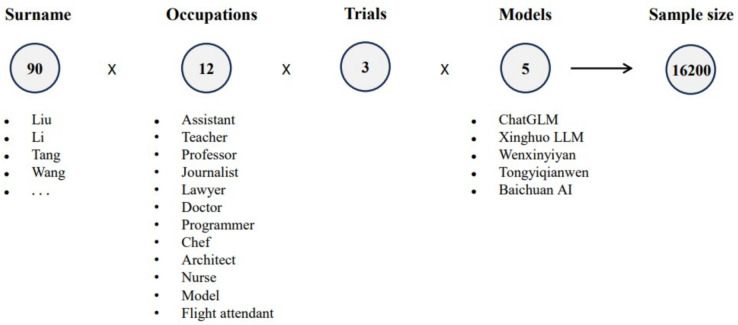
The components of total sample date.

To optimize the model’s input prompts, we carefully selected 90 representative Chinese surnames and 12 occupational categories. The surname list was based on the official ranking published by the Ministry of Public Security, covering more than 80% of the national population and featuring widespread distribution across regions, thus ensuring high representativeness. Regarding occupational categorization, we considered both gender distribution and societal function. Specifically, we included three professions with clear hierarchical structures (Teaching Assistant, Teacher, Professor), three male-dominated professions (Programmer, Chef, Architect), three gender-balanced professions (Journalist, Lawyer, Doctor), and three female-dominated professions (Nurse, Model, Flight Attendant). Although the sample is limited in scope, these occupations span major societal sectors such as education, healthcare, law, media, and service industries, and exhibit distinct gender structures. Therefore, they serve as a reasonable entry point for initial exploration of potential gender and social biases in LLM outputs. In constructing the prompts, we combined surnames with occupations to elicit model-generated personal descriptions containing attributes such as gender, age, educational background, and place of origin. To eliminate contextual carryover, we restarted the dialogue session after each successful generation to ensure output independence. Given the importance of analyzing gender-related probabilities, each surname-occupation pair was repeated three times, and all outputs were collected. As a result, each occupational category yielded a total of 270 data samples. Detailed information on the sample structure is presented in Fig. 3.

It is worth noting that in assessing gender bias, we relied on national labor statistics as a reference baseline. However, for variables such as educational background, age, and regional origin, due to the lack of publicly available occupational distribution data, we focused on observing distributional trends in the generated content rather than quantifying bias with strict statistical comparisons. Our analysis therefore places greater emphasis on identifying qualitative stereotypes manifested in the model outputs, rather than measuring precise levels of deviation.

The following is an example of the specific research conducted:

***Prompt:*** *Please generate an introduction for (Liu) (Assistant), including relevant information such as gender, age, educational background, and place of origin.*

In our study, which focused on the analysis of content generated by C-LLMs, we used a string matching approach using Python to extract information on gender and educational background^36^. Furthermore, we utilized regular expressions to search for and retrieve details on age and place of origin. The extracted information was then outputted in Excel format using the “pandas” library^37^. To ensure the accuracy and effectiveness of subsequent data analysis, we implemented the following preprocessing steps on the collected data: Firstly, we observed that among all C-LLMs, only Tongyiqianwen refused to generate specific content when generating personal introductions for certain professions, explicitly acknowledging its nature as an AI and emphasizing its commitment to privacy protection. For this subset of data, we decided to remove it from our analysis. Additionally, the study found that, except for ChatGLM, the other four C-LLMs exhibited a certain proportion of neutral responses in their output-failing to clearly express personal gender, age, and place of origin. Table 2 summarizes the specific forms of these neutral and biased responses.

**Table 2:** Unbiased and biased examples of personal information generation by C-LLMs related to gender, age, and region. This table presents examples of unbiased and biased outputs generated by C-LLMs in the fields of gender, age, and region, focusing on their performance in unbiased descriptions.

Aspect	Unbiased description	Examples of unbiased outputs	Examples of biased outputs
Gender	he/she, fill in according to the actual situation, man/woman, gender unknown.	Dr. Liu, (man/woman), Age: (can be filled in as needed), holds a Ph.D. degree and is from (a certain region in China). (He/She) has a gentle and kind personality, with extensive clinical experience, earning the trust and praise of numerous patients.	Dr. Liu, (male), (35) years old, was born in the beautiful city of (Suzhou, Jiangsu Province), China. He graduated from a prestigious Chinese medical university with a Doctor of Medicine (M.D.) degree. (He) is committed to providing high-quality medical care to his patients.
Age	fill in according to the actual situation, age unknown, xx years old, unlimited.		
Region	China, a certain province, a certain city, a certain region, could be anywhere, fill in according to the specific situation, area unknown, unlimited.		

**Fig. 4:**
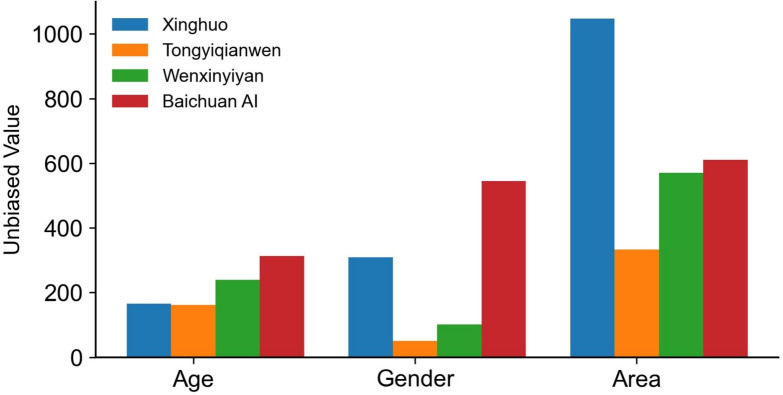
Unbiased statistical representation of age, gender, and region in C-LLMs. A higher value indicates stronger unbiased performance in the corresponding dimension.

We further analyzed neutral or unbiased responses separately. Fig. 4 illustrates the count (y-axis: Unbiased Value, indicating the number of unbiased descriptions as defined by Table 2) of neutral descriptions across gender, age, and region generated by examined C-LLMs. Results show higher regional neutrality across all models, whereas gender and age dimensions exhibit relatively lower unbiased occurrences. Specifically, Xinghuo exhibits the highest frequency of neutral descriptions regarding region, while Baichuan AI performs better in producing unbiased outputs for gender and age. Conversely, Tongyiqianwen generally displays lower unbiased occurrences, reflecting clear disparities among the models. These differences potentially result from variations in training data distributions, socio-cultural contexts, and annotation methodologies. Selecting an appropriate model requires careful consideration of these biases according to specific application scenarios. Future research should emphasize enhancing gender and age neutrality through targeted fine-tuning strategies to improve the overall fairness of C-LLMs.

## Experiments and analyses

This study systematically encoded and quantitatively analyzed key categorical variables, including occupation, sex, age, educational level, and region. We conduct a correlation analysis, and the coefficients reported are Pearson correlation coefficients, with a significance level of $\alpha = 0.05$, to examine the degree of association between occupation and the variables of gender, age, educational level, and region. As shown in Table 3, the analysis reveals statistically significant correlations between occupation and gender, age, and educational background ($p < 0.05$, marked with * in the table), whereas no significant correlation is observed between occupation and region ($p > 0.05$). These results indicate the presence of occupational stereotypes in C-LLMs related to gender, age, and educational attainment, but no evident occupational stereotyping with respect to regional distribution. Based on these findings, the subsequent analysis focuses on examining biases and representational tendencies in C-LLMs across the four dimensions of gender, age, education, and region, while further exploring the occupational stereotypes reflected in gender, age, and educational background.

**Table 3:** Pearson correlation coefficients between occupation and gender, age, education level, and region.

Variables	ChatGLM (Job)	Xinghuo (Job)	Tongyiqianwen (Job)	Wenxinyiyan (Job)	Baichuan AI (Job)
Sex	0.371*	0.278*	0.447*	0.428*	0.325*
Age	-0.516*	-0.465*	-0.442*	-0.510*	-0.404*
Education	-0.649*	-0.646*	-0.650*	-0.583*	-0.659*
Region	-0.023	-0.002	-0.029	-0.018	-0.014

### Bias evaluation

#### Gender perspective

To quantify the degree of gender distribution bias in various professions generated by different models, this study uses Eq.(1) as a metric to evaluate the gender bias of each model between professions. In this context, *B* represents the bias rate, *f* indicates the observed gender proportion, and *g* denotes the expected gender proportion. However, due to the presence of unbiased items and unknown items (the portions of the data that Tongyiqianwen refused to generate) in the collected model generated data, we have optimized and extended Eq.(1) and proposed Eq.(2) for a more accurate calculation of occupational gender bias. Eq.(2) innovatively incorporates the impact of gender unbiased and unknown items on gender proportion within occupation *j* (${u}_{j}$). ${u}_{j}$ cannot accurately reflect the gender distribution. If these neutral or unknown proportions were not considered (directly using ${H}_{ij}$ as the baseline without adjusting for unbiased items), it would distort the bias measurement due to incomplete and imprecise gender proportion calculation. Hence, by incorporating ${u}_{j}$, Eq.(2) effectively excludes the neutral or unknown proportions, ensuring higher accuracy and data integrity in bias measurement. Notably, a positive value indicates that the gender proportion of an occupation in AIGC exceeds its proportion in the labor market, reflecting a preference for that gender. A negative value signifies that the gender representation of an occupation in AIGC falls below its proportion in the labor market, indicating an underrepresentation or bias against that gender. The closer the value is to zero, the smaller the gender bias in that occupation. Furthermore, Eq.(3) is used to calculate the overall bias ${B}_{tj}$ for each occupation, where ${B}_{1j}$ represents the male bias and ${B}_{2j}$ represents the female bias in profession *j*.

1 $$\begin{aligned} B=\frac{f-g}{g} \end{aligned}$$

where *B* represents the relative bias, which measures the deviation between the measured value and the true value. *f* denotes the measured value, *g* represents the expected value.

2 $$\begin{aligned} {B}_{ij}=\frac{{F}_{ij}-{H}_{ij}*\left( 1-{u}_{j}\right) }{{H}_{ij}*\left( 1-{u}_{j}\right) },i=1,2 ; j=1,2,...,12 \end{aligned}$$

where ${B}_{ij}$ represents the degree of bias in occupation *j* toward gender *i* (*i*=1 for male, *i*=2 for female). ${F}_{ij}$ denotes the proportion of gender *i* in occupation *j* within the model-generated content, while ${H}_{ij}$ represents the expected proportion of gender *i* in occupation *j* based on Chinese Statistical Yearbook and employment reports published by authoritative statistical institutions. ${u}_{j}$ refers to the proportion of unbiased and unknown terms related to gender in occupation *j*, reflecting considerations of data completeness and accuracy.

3 $$\begin{aligned} {B}_{tj}=\left| {B}_{1j}\right| +\left| {B}_{2j}\right| ,1=male;2=female \end{aligned}$$

where ${B}_{tj}$ represents the overall gender bias in occupation *j*, ${B}_{1j}$ denotes the absolute bias for males in occupation *j*, and ${B}_{2j}$ denotes the absolute bias for females in occupation *j*.

**Fig. 5:**
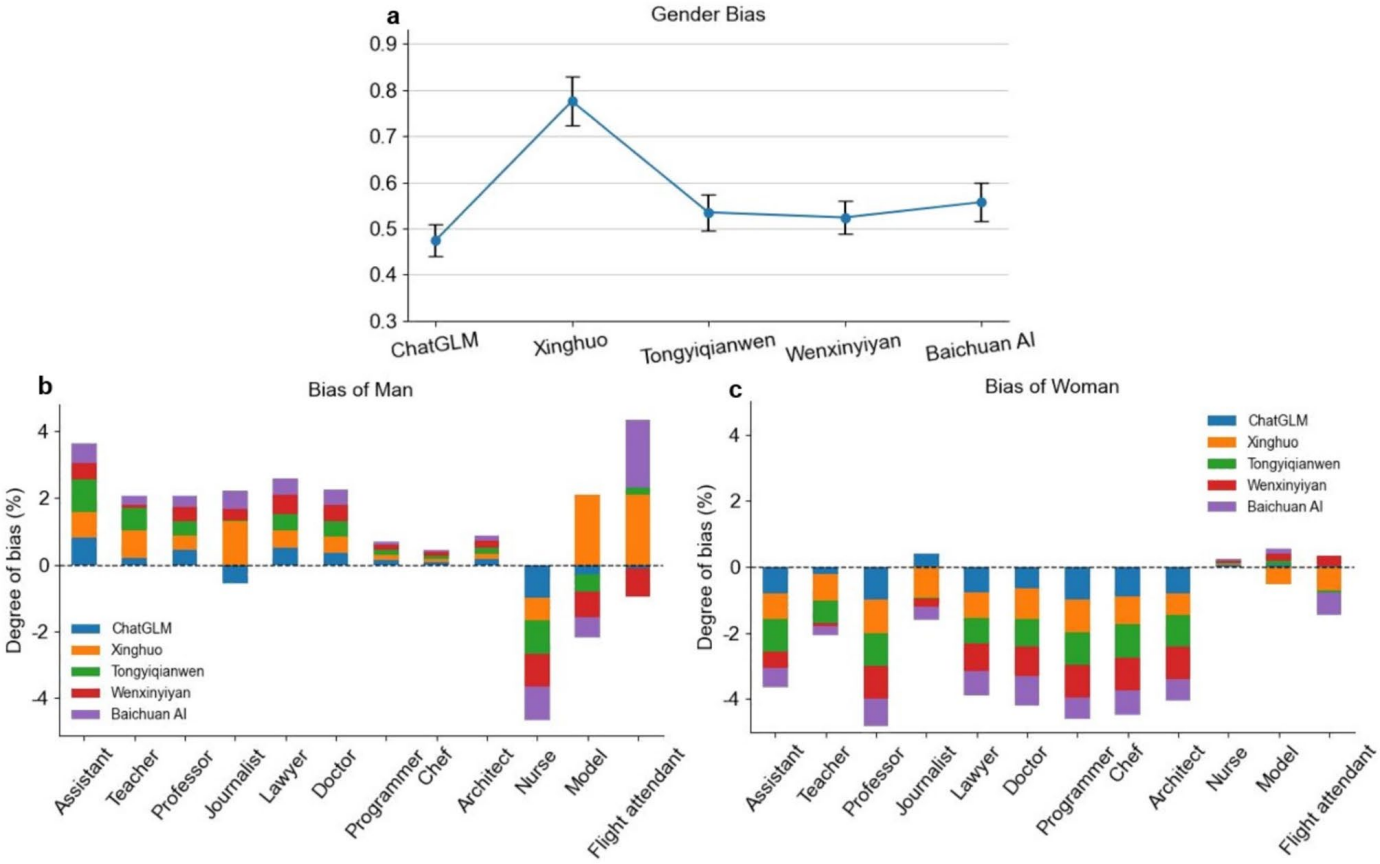
Gender bias in occupational representation by C-LLMs. (**a**) Total gender bias: The comparison of the average overall gender bias across all occupations for each examined C-LLMs. Error bar indicates 95% confidence interval. (**b**) Bias of Man: A positive value indicates a bias toward men in the given profession, while a negative value suggests an underrepresentation of men in that profession. (**c**)Bias of Woman: A positive value indicates a bias toward women in the given profession, while a negative value suggests an underrepresentation of women in that profession.

Based on the calculations from Eq.(3), we have plotted the average overall bias for each examined C-LLMs, as shown in Fig. 5a. The data indicate that ChatGLM performs the best among the models, with an overall bias of 0.474. Wenxinyiyan, Tongyiqianwen, and Baichuan AI follow, with overall biases of 0.524, 0.535, and 0.557, respectively. Although the latter two exhibit relatively higher levels of bias, they still perform better than the worst model, the Xinghuo Large Model. The Xinghuo Large Model shows the highest overall bias, reaching a significant level of 0.776. This could have implications for the fairness of large language models in areas such as recruitment, career planning, and education.

Figures 5b and 5c respectively illustrate the male and female occupational gender biases exhibited by different C-LLMs. Our findings reveal a clear presence of occupational stereotypes: The examined C-LLMs generally exhibit a preference for males when describing class-based occupations, male-dominated professions, and occupations with a relatively balanced gender distribution (as evidenced by positive values in Fig. 5b and negative values in Fig. 5c). This gender preference only disappears when describing occupations predominantly held by women, shifting instead to a preference for females (negative values in Fig. 5b and positive values in Fig. 5c). For instance, in the nursing profession, all models consistently demonstrate a strong preference for women. Notably, in female-dominated occupations such as modeling and flight attendants, Xinghuo, Tongyi Qianwen, and Baichuan AI still exhibit varying degrees of male preference. This observation suggests that these three models require further optimization and refinement to improve stability and mitigate biases in their generated content.

#### Age perspective

In the evolutionary process of careers, the close correlation between vocational choices and individual age forms a significant dimension for research. Individuals in different age groups exhibit distinct characteristics in their career choices and development cycles. This paper elaborates on the age distribution in the content generation processes of C-LLMs, aiming to uncover the underlying patterns. Table 4 presents a descriptive statistical analysis of the age distribution in the outputs of each model. After eliminating the unbiased and unknown elements from the models, we ensured that the effective data size for each C-LLMs was no less than 2,748. The study found that the average age of the content generated by the models ranges from 35 to 40 years old, coinciding with the mature stage of a career. This finding reflects the model’s emphasis on the core strength of the vocational lifecycle during content construction. It is noteworthy that Tongyiqianwen exhibits the highest standard deviation in age distribution, indicating extensive coverage and diversity in the age span of generated content. In contrast, Xinghuo, with the lowest standard deviation of 7.183, highlights a high concentration of its output content at the age level. This may be attributed to the model’s preference for data from a particular age group during the training process or the limitations of its generation strategies.

**Table 4:** Descriptive statistics of age in content generated by examined models.

Statistics	ChatGLM	**Xinghuo**	Tongyiqianwen	Wenxinyiyan	Baichuan AI
N	3240	3075	2748	3000	2929
Min	23	18	25	20	20
Max	55	68	58	58	65
Mean	35.19	36.57	38.64	38.12	38.37
Standard Deviation	7.913	7.183	8.586	7.764	8.003
Skewness	1.039	0.531	0.362	0.490	0.303
Kurtosis	0.454	-0.078	-0.832	-0.419	-0.665

Further analysis reveals that the ChatGLM model shows significant positive skewness, favoring younger age groups, while the Baichuan AI model, with skewness near zero, maintains a balanced age distribution. This discrepancy may be due to differences in the design of the model architecture, the diversity of training datasets, and the strategies to control randomness in content generation algorithms. The variance in skewness metrics offers a unique perspective on the preference of different C-LLMs algorithms for age characteristics. Moreover, most models display negative kurtosis values, suggesting that these models tend to avoid outputs at extreme age values during content generation. This characteristic may be influenced by multiple factors, such as the design of the loss function of the model, regularization strategies, and optimization objectives. In-depth analysis of the age distribution differences among these models can facilitate further optimization, enabling the generated content to better align with the needs of specific application scenarios.

**Fig. 6:**
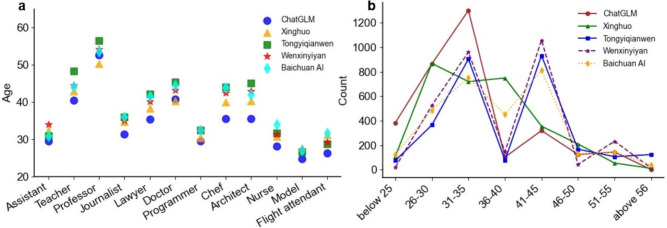
Age bias in occupational representation by C-LLMs. (**a**) Age Distribution Across Occupations: The vertical axis represents the average age predicted by the model for each occupation. (**b**) Age Group Count: The vertical axis represents the frequency of occurrences for each age group in the model’s predictions.

As illustrated in Fig. 6a, C-LLMs generally display a consistent trend in predicting the mean age levels for various occupations. Specifically, for occupations such as professors, teachers, and doctors, the predicted average age by the models tends to be relatively high (40-60 years old). This result can be attributed to the fact that these occupations typically require extensive professional experience and long-term accumulation of expertise. In contrast, for occupations such as models and flight attendants, the predicted average age is lower (20-30 years old), which can be explained by the preference for younger professionals in these fields.

Further analysis of the data presented in Fig. 6b reveals significant fluctuations in the models’ prediction results across different age groups. Notably, while the Xinghuo model peaks at the 26-30 and 36-40 age ranges, other models reach their predictive peaks within the 31-35 and 41-45 age brackets, this discrepancy may stem from differences in the training data and algorithmic processing methods used by various C-LLMs. Predictions for those under 25, between 36-40, and over 46-50 are markedly lower. This result indicates that C-LLMs tend to favor middle-aged individuals ( between 30 and 45 years old) when outputting age-related characteristics for occupations, with notably less representation for extremely young or older groups.

From a perspective of social impact, such bias can exacerbate stereotypes about young and old individuals, reduce their representation in application scenarios, and consequently affect the completeness and fairness of the model. Therefore, to enhance the models’ generalization capabilities and promote fairness in applications, it is imperative to strive for greater diversity in training datasets. This includes encompassing a wider range of occupational categories, age levels, and cultural backgrounds. Ensuring this diversity will allow the C-LLMs to more accurately reflect and serve the needs of all strata of society.

#### Education perspective

**Fig. 7:**
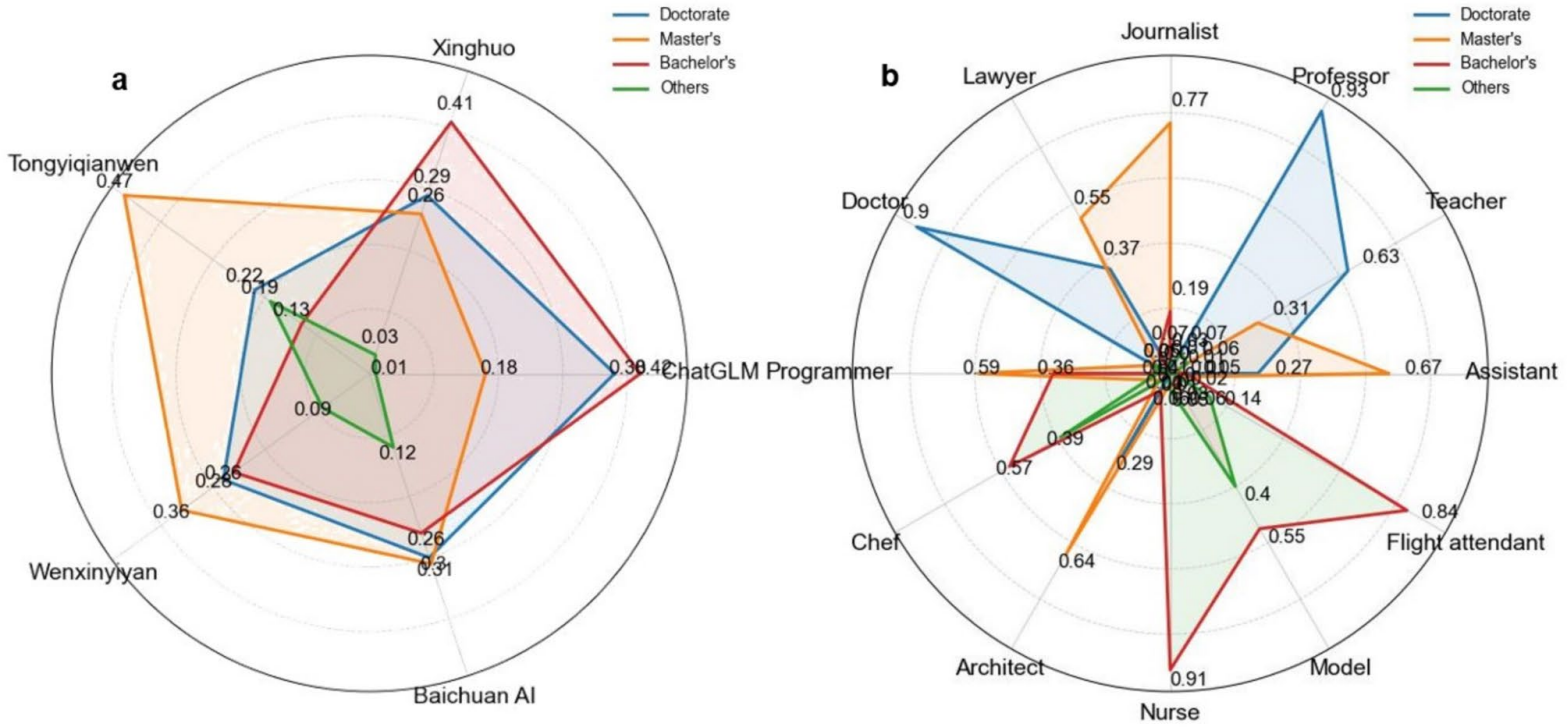
Educational background bias in occupational representation by C-LLMs. (**a**) Educational Bias Across Models: This figure illustrates the educational preferences exhibited by different C-LLMs. (**b**) Educational Distribution Across Occupations: This figure presents the proportion of different educational levels associated with various professions in AIGC.

Fig. 7a illustrates the distribution of educational backgrounds in content generated by different models. The analysis reveals a general tendency across all models to produce individuals with bachelor’s degrees or higher, suggesting that these models are more likely to associate professions with higher levels of education. Specifically, ChatGLM generates the highest proportion of doctoral-level profiles, while Tongyiqianwen and Wenxinyiyan produce more individuals with master’s degrees. ChatGLM and Xinghuo lead in generating bachelor’s degree holders. In contrast, the Baichuan AI model shows a more balanced distribution across bachelor’s, master’s, and doctoral levels. These differences in the distribution of educational background among the models reflect their applicability at various educational levels, providing researchers with a basis for selecting appropriate models that better match the educational background and knowledge needs of the users.

Furthermore, to deeply explore the distribution of educational backgrounds within professional fields, this study categorizes occupations and conducts a statistical analysis of the educational distribution in content generated by five C-LLMs, as shown in Fig. 7b. The data reveal a distinct stratification of educational levels across different professions. Doctoral degrees are mainly focused on professors and doctors; professions like journalists and architects have a high percentage of master’s degrees; bachelor’s degrees are predominant among nurses and flight attendants, while occupations such as chefs and models have a higher proportion of degrees at the bachelor’s level or below. These characteristics of educational distribution across occupations suggest that C-LLMs, to some extent, perpetuate societal stereotypes and biases regarding the educational levels of professions. In other words, professions with a higher proportion of individuals holding advanced degrees are often perceived as more advantageous or higher-tiered. This phenomenon is manifested in the output of large language models, revealing a new dimension of professional bias: the potential preference and reverence for professions associated with higher education. This analysis helps us better understand the multiple factors contributing to differences in educational distribution and provides a research pathway for evaluating the effectiveness of C-LLMs and the dynamic impact on various professions. It holds significant importance in promoting model fairness and reducing bias.

#### Regional perspective

**Fig. 8:**
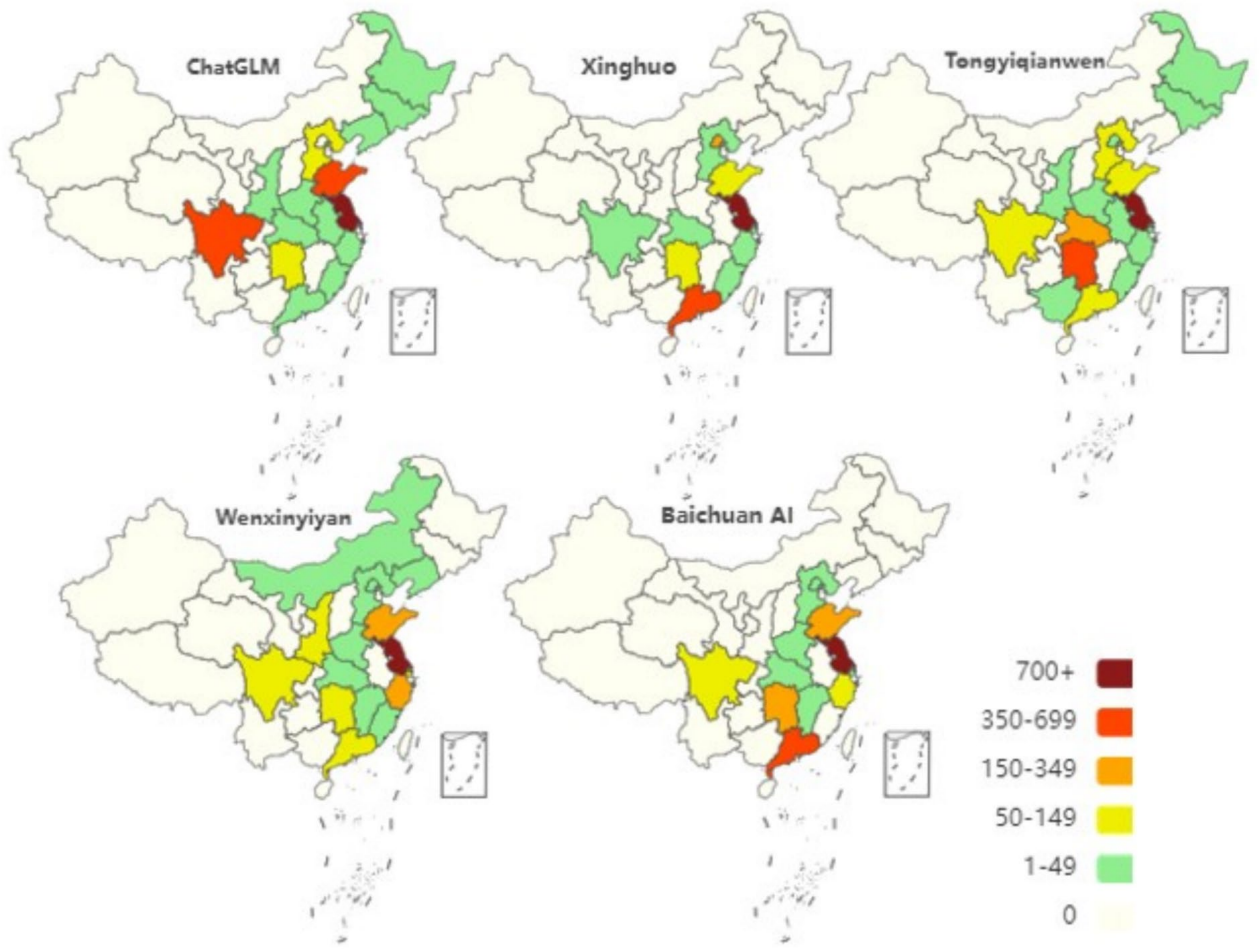
Regional distribution in the content generated by C-LLMs. Map lines delineate study areas and do not necessarily depict accepted national boundaries.

Fig. 8 illustrates the distribution characteristics of various Chinese provinces of origin involved when different C-LLMs generate introductory content. This study aims to delve into the coverage and application status of various C-LLMs within different regions of China by examining this distribution.

As observed from Fig. 8, the five C-LLMs exhibit significant regional heterogeneity in terms of application and data distribution across Chinese regions. Firstly, concerning the breadth of regional coverage, the Xinghuo model has the narrowest scope, covering only 11 regions. In contrast, the Tongyiqianwen model has the most extensive regional coverage, reaching up to 17 regions. This comparison suggests that the Xinghuo model requires further optimization in terms of its generalization capability. Secondly, regarding the depth of regional coverage, the performance of each model varies. Specifically, all models exhibit the highest data density in Jiangsu Province, with Sichuan, Guangdong, Shandong, and Hunan provinces also demonstrating relatively high values in some models. This indicates that these provinces are the primary areas for the application or data sourcing of the models. Furthermore, moderate values are observed in provinces such as Zhejiang, Liaoning, Hubei, and Hebei, suggesting that although these provinces have a certain degree of representativeness in the application of the models, their influence remains somewhat limited compared to regions with higher values. Notably, provinces in the west, south and northeast, such as Xinjiang, Qinghai, and Yunnan, exhibit low data density or even a complete absence of data in most models. This observation directly reflects the inadequacies in the data collection and application coverage in these regions, highlighting a significant bias in the regional coverage of the models.

In total, Fig. 8 not only provides a visual representation of the uneven geographical coverage and application of the five C-LLMs, but also deeply reveals the potential regional bias issues that may exist in their development and application. If such biases are not addressed, they can lead to the neglect of specific regional cultures, linguistic characteristics, and user needs, thereby affecting the overall performance and universality of the models. Therefore, future developers of C-LLMs should prioritize the diversity and balance of data sources. By optimizing data collection strategies and algorithm design, they should strive to reduce and eliminate regional biases, with the aim of constructing a more comprehensive, fair, and efficient intelligent language processing system.

### Bias mechanism analysis

**Fig. 9:**
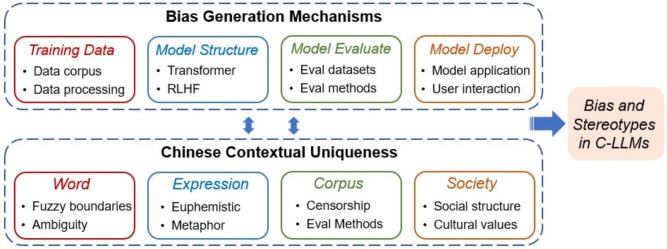
Analytical framework for bias and stereotype generation mechanisms in C-LLMs. This figure illustrates the interaction between four critical components-training data, model architecture, model evaluation, and model deployment-and the unique characteristics of the Chinese context, which collectively lead to bias and stereotype generation in C-LLMs.

The biases and stereotypes regarding gender, age, educational background, and regional characteristics exhibited by C-LLMs in generating personal profiles are not incidental errors. Instead, they are systemic results arising from interconnected influences throughout the model lifecycle. From training data collection and model training mechanisms to evaluation designs and deployment interactions, decisions made at every stage may either introduce or amplify biases. Furthermore, the distinctive features of the Chinese language and its sociocultural environment further exacerbate the complexity and subtlety of these biases. As illustrated in Fig. 9, this section will thoroughly examine the mechanisms by which biases are produced in C-LLMs, emphasizing four critical stages within the specific context of the Chinese language.

#### Bias introduction at the training data stage

Training data forms the foundational layer of knowledge representation in LLMs, and its quality and representativeness play a decisive role in shaping the fairness and diversity of model outputs. In the case of C-LLMs, the pre-training corpora often exhibit pronounced structural biases^38^. Existing studies indicate that approximately 65-80% of the pre-training data for C-LLMs come from high-traffic platforms such as social media, news outlets, and digital publications. These sources disproportionately reflect the linguistic styles and sociocultural ideologies of urban, young, and highly educated users, while significantly underrepresenting marginalized voices, minority dialects, and cross-cultural expressions^39^. This imbalance leads C-LLMs to overfit mainstream narratives, reducing their ability to generalize across diverse demographic and cultural contexts.

Moreover, widespread societal biases embedded in the source data, such as rigid gender norms, regional stereotypes, and occupational hierarchies, are easily internalized by the model if not explicitly mitigated. For example, in training corpora, terms like “engineer” and “leader” frequently co-occur with male pronouns, whereas roles such as “nurse” or “secretary” often align with female references^40^. Furthermore, data collection practices also amplify these structural biases. In pursuit of scale and efficiency, developers often prioritize high-engagement platforms, which inherently filter for popular discourses and trending topics. As a result, marginalized narratives are not only under-collected but may also be systematically removed during preprocessing. Human annotators or content moderation algorithms frequently exclude politically sensitive or non-mainstream content, forming the so-called “information silence zones”^41^. This selective representation in training data becomes the root cause of bias propagation in downstream outputs.

#### Bias amplification in model training mechanisms

C-LLMs are primarily trained to minimize prediction errors, a process that inherently biases the learning dynamics toward dominant linguistic patterns and high-frequency co-occurrences. This optimization objective causes models to preferentially internalize mainstream expressions while neglecting rare or marginalized language features^42^. The Transformer architecture, particularly its attention mechanism, further reinforces these tendencies by amplifying associations between frequently co-occurring tokens. For instance, the CORGI-PM corpus reveals that occupational terms like “programmer” are predominantly associated with male pronouns, reflecting societal stereotypes embedded in training data^43^. Similarly, Zhao et al. demonstrate that large language models tend to associate technical professions with male identities, further perpetuating gendered assumptions^44^. These associations are not neutral byproducts of statistical modeling, but rather reflections of deeply embedded societal norms present in the training data.

Moreover, the dynamics of model optimization exacerbate these initial biases. An internal technical report on ERNIE 3.0 reveals that the gradient variability of gender-related parameters dropped by 73% during the early training stages, indicating rapid convergence on socially dominant associations^45^. This suggests that models tend to lock in biased correlations early in the training process due to their optimization trajectory. Without regularization mechanisms or counter-bias signals, these early-formed associations become effectively “frozen” within the model. In addition, instruction fine-tuning and RLHF processes introduce further subjectivity through human feedback^46^. When feedback data lack demographic diversity or are filtered through unbalanced annotator perspectives, it risks reinforcing the very stereotypes the model was meant to avoid. Therefore, both the architecture design and optimization procedure of C-LLMs play pivotal roles in amplifying initial corpus biases, transforming subtle data imbalances into persistent output patterns.

#### Bias concealment in the evaluation stage

The evaluation stage is intended to ensure quality control and fairness checks during the development of C-LLMs. However, current evaluation practices often fail to uncover embedded structural biases due to limitations in both assessment metrics and dataset construction. Mainstream metrics such as accuracy, BLEU score, and perplexity focus on aggregate semantic performance but are insufficient to detect disparities across demographic subgroups^47^. Consequently, they may mask performance disparities among different social groups. Recent empirical studies reinforce these concerns. For example, the C-EVAL benchmark shows that only 0.7% of its test cases involve minority languages, and 99.2% of samples exclusively use standardized Mandarin^48^. This severe lack of linguistic and cultural diversity constrains the benchmark’s ability to evaluate fairness in non-mainstream contexts. More importantly, traditional metrics struggle to detect discriminatory outputs from models. The recently proposed BEATS bias evaluation framework reveals that conventional single-metric indicators, such as accuracy, exhibit significant blind spots in detecting discriminatory outputs from large language models, often obscuring biased behaviors toward specific demographic groups^49^.

Additionally, automated evaluation tools exhibit limited effectiveness. These tools struggle to capture implicit meaning, metaphorical usage, and polysemous constructs, especially in Chinese, where contextual cues are subtle and grammar is non-explicit. Koo et al. introduced the Cognitive Bias Benchmark for LLMs as Evaluators (CoBBLEr), and found that around 40% of model judgments showed explicit biases. Meanwhile, the Rank-Biased Overlap (RBO) between model outputs and human bias assessments was only 49.6%, revealing significant inconsistency^50^. This suggests that LLMs, even when used in evaluation, may reproduce the very biases they aim to detect, leading to a recursive bias loop within the evaluation stage. Furthermore, human evaluators may also unconsciously introduce subjective biases due to their homogeneous backgrounds or experiential tendencies. Prior studies show that crowd annotators tend to assign biased ratings based on personal experience, especially when dealing with socially sensitive tasks^51^. In summary, existing evaluation systems contain substantial bias concealment across metric design, corpus composition, evaluation tool efficacy, and evaluator subjectivity. Consequently, models that perform well under standardized evaluations may still pose considerable risks of generating unfair outcomes in real-world scenarios.

#### Bias reproduction in deployment and interaction

The deployment of C-LLMs in real-world environments establishes a feedback loop through which social biases may be amplified and reinforced^52,53^. Increasingly, C-LLMs are applied in socially sensitive domains such as education, recruitment, healthcare, and legal services. In the absence of robust bias mitigation strategies, these models may influence users’ perceptions and decisions in subtle but consequential ways. Empirical studies have shown that LLMs trained on biased datasets tend to reproduce occupational stereotypes. A recent audit of model-generated job descriptions found that technical roles were assigned male pronouns over 85% of the time, whereas caregiving and nursing roles were disproportionately associated with female pronouns^54^. When embedded into algorithmic decision-making systems, such biased outputs risk normalizing discriminatory assumptions in hiring practices and career guidance applications.

Moreover, The interactive nature of C-LLMs compounds these risks. These models are highly responsive to user prompts and adept at adapting to linguistic nuances, which enhances usability but also increases vulnerability to prompt-induced bias. When users submit biased or leading queries, models frequently affirm the underlying assumptions without critique, thereby fostering a “bias echo chamber” over repeated interactions. Prompt-based experiments confirm this phenomenon, showing that gender-biased questions significantly elevate stereotypical completion rates across mainstream LLMs^55^. An additional concern is the lack of transparency and explainability in most deployed C-LLMs. Operating as black-box systems, these models offer limited interpretability for users and developers, making it difficult to trace, understand, or correct biased outputs. Research on AI explainability has shown that low system transparency diminishes user trust and impedes timely detection of harmful outputs at the point of use^56^. In sum, the combination of high adaptability, low explainability, and wide deployment in socially impactful contexts makes C-LLMs vulnerable to reinforcing harmful social biases in practice. Without systemic safeguards, deployment itself becomes a vector of bias reproduction.

#### Bias reinforcement in the Chinese linguistic context

The unique linguistic structure and sociocultural characteristics of the Chinese language create fertile ground for the reinforcement of biases in C-LLMs. This reinforcement arises primarily from linguistic ambiguity, nuanced expression styles, selective silencing in corpus construction, and distinctive cultural contexts. Firstly, unlike Indo-European languages, Chinese lacks explicit grammatical markers for gender, number, or tense, making semantic disambiguation highly context-dependent. As a result, when identity attributes such as profession or social status are involved, models often resolve ambiguity using learned statistical associations-frequently defaulting to dominant norms. For example, Chaturvedi et al. found that LLMs associate male-related terms (e.g., “ability to work under pressure”) in job postings with a higher probability of recommending male candidates, while linking female-related terms (e.g., “detail-oriented and patient”) to a higher likelihood of recommending female candidates^54^. Consequently, even in the absence of explicit gender indicators, these models internalize and perpetuate entrenched gender stereotypes in occupational contexts. Secondly, Chinese discourse is characterized by high-context communication, where indirectness, euphemism, and metaphor are common rhetorical strategies. These features complicate bias detection and mitigation. Expressions such as “leftover woman” or “strong woman” may appear neutral on the surface but encode deeply entrenched gender hierarchies and social expectations. Most C-LLMs lack sufficient sociolinguistic grounding to distinguish between literal and connotative meaning, thus replicating these implicit biases in output generation^57^.

Moreover, Bias reinforcement is further compounded by selective silence in corpus construction. Empirical audits of Chinese web corpora have revealed substantial underrepresentation of minority discourses, including content related to gender equality, LGBTQ+ rights, and regional identities^48^. Such omissions not only skew the training distribution but also reinforce dominant ideological narratives. Lastly, the corpus implicitly embodies societal structures and cultural norms, including traditional gender roles, educational elitism, and regional hierarchies, subtly reinforcing these biases within models. Repeated exposure to such structural biases leads models to adopt them as linguistic norms, translating societal inequalities into algorithmic outputs. Therefore, bias reinforcement in C-LLMs is not merely a technical artifact but a sociolinguistic consequence of modeling a high-context, ideologically stratified language without appropriate cultural calibration.

## Discussion

In this study, we proposed a structured approach to evaluating bias in Chinese large language models (C-LLMs) through the generation of personal profile texts. Our findings indicate that C-LLMs consistently exhibit traces of bias and social stereotypes inherited from their training data. First, with regard to neutrality, all four C-LLMs examined-Xinghuo, Tongyiqianwen, Wenxinyiyan, and Baichuan AI-demonstrate varying degrees of restraint in generating specific demographic attributes such as gender, age, and education. Among them, only Tongyiqianwen clearly adopts a privacy-preserving stance by refusing to produce such content, highlighting notable differences in model behaviors that users should consider when selecting a model for specific applications. Second, in the bias analysis across four dimensions-gender, age, education, and regional background-the models reveal consistent patterns of stereotypical representations. Male bias is prevalent across most occupations, with the default gender often assumed to be male; this bias diminishes only in female-dominated professions and occasionally reverses. Age preferences cluster around the ranges of 31-35 and 41-45, except in cases where professional norms dictate otherwise. Educationally, the models tend to favor profiles with at least a bachelor’s degree, though the degree distribution varies by profession. Regional bias is evident in the overrepresentation of candidates from China’s eastern and central provinces, with limited reference to western and northern regions.These biases reflect systemic outcomes shaped by multiple stages in the model lifecycle: data collection during pre-training, algorithmic configurations during training, evaluation criteria during assessment, and user interaction during deployment. Furthermore, the inherent cultural and linguistic characteristics of the Chinese language contribute to the subtlety and complexity of bias expression in C-LLMs. Understanding these mechanisms is essential for interpreting model outputs and designing more equitable AI systems.

This study has several practical contributions. First, we have shown that C-LLMs exhibit a certain degree of bias with respect to gender, age, educational level, and other factors. This finding provides a reference for identifying and correcting biases within the models, contributing to the fairness of generated content, and enhancing its acceptance and usage across various applications. Second, By addressing both the micro-level modeling mechanisms and the macro-level linguistic structures, this study systematically elucidates the multifactorial mechanisms underlying bias generation in C-LLMs. It offers structural insights for future efforts in bias detection and mitigation mechanism design, and holds promising application prospects. Third, with the advancement of artificial intelligence technology, regulatory bodies are imposing stricter requirements on the fairness and transparency of LLMs. Investigating model bias aligns with relevant regulations and standards, and it contributes to the further development of natural language processing technologies, leading to more efficient and reliable model design and application.

Notwithstanding the above, this study has several limitations that future research should address. First, the occupational scope covered here is somewhat limited, omitting emerging professions and highly specialized technical roles. Future research should broaden the occupational classification systems and refine the research scope to more accurately capture dynamic labor market changes. Second, this study analyzed only five C-LLMs and excluded models developed in other languages. Future studies could include additional models, such as OpenAI’s ChatGPT, and conduct comparative assessments to explore how biases differ among models across various domains. Moreover, while this research applied a strict definition for assessing gender bias the analysis of age, education, and regional biases relied on descriptive methods due to a lack of baseline data. This inconsistency limits bias measurement precision. Future research should collect or integrate detailed demographic benchmark data to facilitate more systematic and comparable bias evaluations. Finally, this study primarily employed summary-generation tasks to analyze biases in C-LLM outputs, which may introduce subjectivity or ambiguity. Future research should consider alternative task types, such as disambiguation tasks^17^, to better define and quantify potential biases in these models. As research in this field progresses, C-LLMs are expected to evolve toward greater equity and efficiency.

## Data Availability

The data and code necessary to replicate this project are available at https://data.mendeley.com/datasets/n54mks9dzx/1.
